# Heparanase modulates the prognosis and development of *BRAF* V600E-mutant colorectal cancer by regulating AKT/p27Kip1/Cyclin E2 pathway

**DOI:** 10.1038/s41389-022-00428-0

**Published:** 2022-09-21

**Authors:** Mengling Liu, Xiaojing Xu, Ke Peng, Pengcong Hou, Yitao Yuan, Suyao Li, Xun Sun, Zhongyi Shi, Jiayu Zhang, Yu Dong, Qing Liu, Luoyan Ai, Li Liang, Lu Gan, Qihong Huang, Yiyi Yu, Tianshu Liu

**Affiliations:** 1 Department of Medical Oncology, Zhongshan Hospital, Fudan University, Shanghai, 200032 China; 2 Department of General Surgery, Zhongshan Hospital, Fudan University, Shanghai, 200032 China; 3 Department of Clinical Sciences, Zhongshan Hospital, Fudan University, Shanghai, 200032 China; 4 Cancer Center, Zhongshan Hospital, Fudan University, Shanghai, 200032 China; 5 Shanghai Respiratory Research Institute, Zhongshan Hospital, Fudan University, Shanghai, 200032 China; 6 Center of Evidence-based Medicine, Fudan University, Shanghai, 200032 China

**Keywords:** Cancer genetics, Gastrointestinal cancer

## Abstract

*BRAF* V600E-mutant colorectal cancer (CRC) is a rare subtype of colorectal cancer with poor prognosis. Compelling evidence indicates that the heparanase (*HPSE*) gene has multiple functions in cancer, however, its role in *BRAF* V600E-mutant CRC remains elusive. Differentially expressed genes between *BRAF* V600E-mutant and wild-type patients were explored by analyzing public data from The Cancer Genome Atlas and the Gene Expression Omnibus. Clinical samples of 172 patients with *BRAF* V600E-mutant CRC diagnosed at Zhongshan Hospital Fudan University were collected. Overall survival was analyzed using Kaplan–Meier curves and Cox regression models. Cell models and xenografts were utilized to investigate the effect of *HPSE* on tumor proliferation. *HPSE* was significantly highly expressed in the *BRAF* V600E-mutant group. High *HPSE* expression level was independently associated with inferior survival in the *BRAF* V600E-mutant cohort. *HPSE* knockdown impeded tumor proliferation of *BRAF* V600E-mutant CRC cells in vitro and in vivo. Mechanistically, *HPSE* silencing arrested cell cycle in G0/G1 phase by downregulating Cyclin E2 expression via the AKT/p27Kip1 pathway. These findings support a role for *HPSE* in promoting *BRAF* V600E-mutant CRC progression, which suggests it holds great promise as a prognostic biomarker and a potential therapeutic target for the aggressive CRC subtype.

## Introduction

Colorectal cancer (CRC) is among the most common cancers and the leading cause of cancer-related deaths worldwide. As a highly heterogeneous disease, evidence shows that molecular classification plays a major role in CRC management [1]. *BRAF* V600E-mutant subtype only accounts for about 10% in CRC [2], which has received much attention from researchers in recent years. BRAF protein with V600E mutation has been demonstrated to promote tumor development by directly activating the MEK/ERK signaling pathway as a monomer independent of RAS [3]. Clinical evidence indicates that *BRAF* V600E-mutation is associated with significantly poor survival in CRC [4–6]. Compared to *BRAF* wild-type CRC, V600E-mutant patients exhibit decreased differentiation and advanced stage [7].

However, complex molecular biological mechanisms underlying the clinical features of *BRAF* V600E-mutant CRC are yet to be fully elucidated, and available treatment options for this subtype are not effective [8]. There is a high unmet need in the clinic for better stratifying and treating *BRAF* V600E-mutant CRC patients. Although researchers have attempted to explore potential therapeutic targets by dissecting gene expression patterns in *BRAF* V600E-mutant CRC, some promising genes are underexplored [9–11].

Analysis in the current study showed that heparanase (*HPSE*) is one of the significantly differentially expressed genes (DEGs) in *BRAF* V600E-mutant CRC patients. This gene encodes HPSE protein which performs multiple functions independent of its enzymatic activity in tumor cells and the tumor microenvironment [12, 13]. Several studies on various cancers have demonstrated the association of *HPSE* in tumor progression and resistance to treatment via numerous mechanisms, including promotion of signal transduction, regulation of gene transcription, cell autophagy, extracellular matrix remodeling, and modulation of the tumor microenvironment [14, 15]. Inhibitors targeting *HPSE* also have a high potential in managing various cancers [16]. Recently, HPSE was revealed to potentially promote growth, proliferation, and liver metastasis of SW480 and SW620 CRC cells by activating the p38/MMP1 axis [17]. Elsewhere, Syndecan-1-mediated regulation of HPSE was reported to influence cell invasion, stemness, and chemotherapy sensitivity in CACO2 CRC cells [18]. Geetha and colleagues also revealed that BRAF kinase activation upregulated *HPSE* expression by regulating its promoter activity [19]. However, these previous studies did not investigate the function and mechanism of *HPSE* in *BRAF* V600E-mutant CRC.

Therefore, we herein attempted to explore the role of *HPSE* in *BRAF* V600E-mutant CRC. First, we identified a significant differential expression of *HPSE* in *BRAF* V600E-mutant CRC versus wild-type (including *KRAS*-mutant and *KRAS*/*BRAF* wild-type) CRC using integrated data, and performed survival analyses in a retrospective clinical cohort. Results revealed a significant and independent prognostic role for HPSE in patients with *BRAF* V600E-mutant CRC. Furthermore, we investigated the role and mechanism of *HPSE* in *BRAF* V600E-mutant CRC in vitro and in vivo. According to the analysis, HPSE promoted cell proliferation by regulating the cell cycle in CRC cells carrying *BRAF* V600E mutation via the AKT/p27Kip1 pathway.

## Results

### *HPSE* is differentially expressed between *BRAF* V600E-mutant and wild-type CRC

**Fig. 1:**
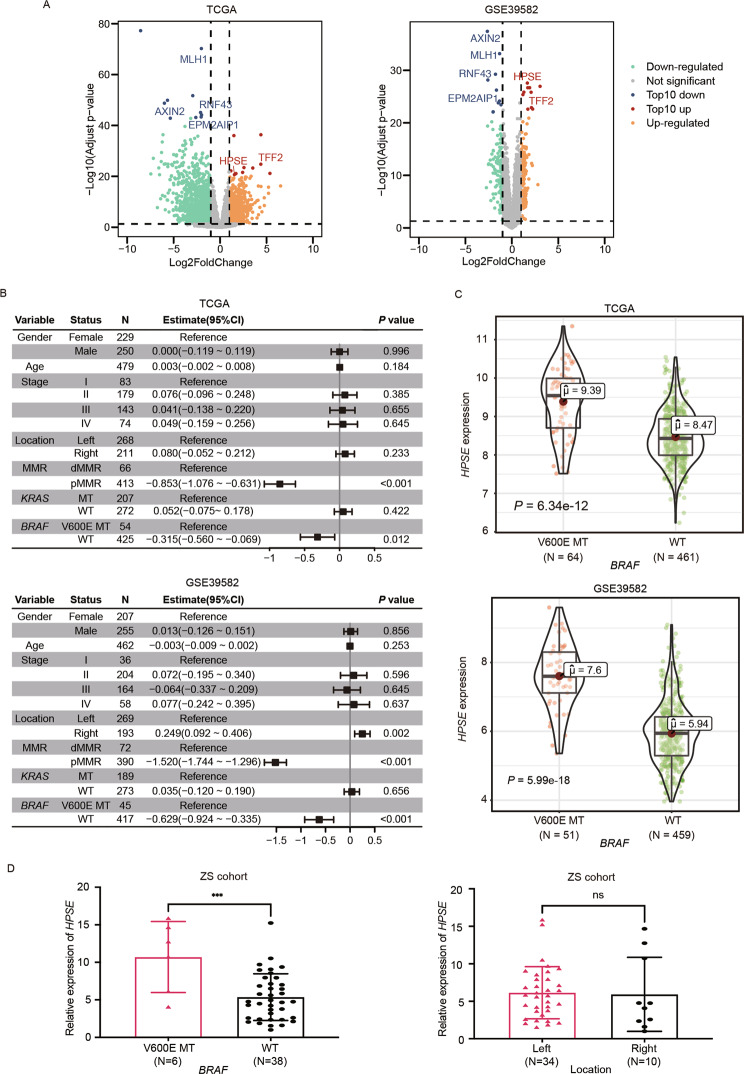
*HPSE* expression level is significantly higher in *BRAF* V600E-mutant compared to BRAF wild-type patients. **A** Volcano maps of differentially expressed genes between *BRAF* V600E-mutant and wild-type colorectal cancer patients in the TCGA dataset (left) and GSE39582 dataset (right). Dots represent genes. Significantly upregulated and downregulated genes, based on a cutoff fold change of 2 and an adjusted *P*-value at 0.05, are colored in orange and green, respectively. The top 10 upregulated and downregulated genes are colored in red and blue, respectively. Common six genes were labeled with gene symbols. The ggpubr and ggthemes R packages were used to generate the map. **B** Multiple linear regression analysis of *HPSE* expression levels in public datasets. Samples with missing values were removed. *F* test *P* < 0.001 in TCGA and GSE39582 datasets. Estimate (blocks in the center) and 95% confidence intervals (Whiskers of error bars) are shown. CI confidence intervals. **C** Expression levels of *HPSE* in *BRAF* V600E-mutant and *BRAF* wild-type CRC patients based on public data. Dots in violin plots represent samples. Centerlines indicate median, red dots indicate the mean, box plots indicate the quartiles, and bars indicate 95% confidence intervals. Violin plots were generated using ggplot2 package and ggstatsplot package. A two-sided Welch’s *t*-test was used to determine *P* values. **D** Higher *HPSE* expression level in *BRAF* V600E-mutant CRC verified using frozen tissue of CRC samples from Zhongshan hospital. Locations of the primary tumor were unbalanced between *BRAF* V600E-mutant and wild-type samples (Table S2); however, no difference was reported in *HPSE* expression level between different primary tumor sites. Data are presented as mean ± standard deviation. The student’s *t*-test was used to calculate *P* values. ***P < 0.001, ns *P* > 0.05.

Analysis of DEGs was performed using transcriptional sequencing data of 525 patients from the TCGA dataset and 510 patients from the GSE39582 dataset. All patients (*N* = 1035) were diagnosed with colorectal cancer and the mutation status of *BRAF* was determined. Basic characteristics of the patients and differences between *BRAF* V600E-mutant and wild-type groups are presented in Table S1. A total of 1974 DEGs and 264 DEGs were identified in TCGA and GSE39582 datasets, respectively, using a threshold of a fold change of 2 and an adjusted *P*-value of 0.05 (Fig. 1A). Top 10 upregulated or downregulated DEGs in both datasets were sorted based on the adjusted *P*-value, then six common top genes were determined (Fig. S1A). Expressions of *HPSE* and *TFF2* were upregulated significantly in *BRAF* V600E-mutant CRC. On the other hand, expressions of *AXIN2*, *MLH1*, *RNF43*, and *EPM2AIP1* were significantly downregulated in *BRAF* V600E-mutant CRC, versus either *KRAS/BRAF* wide-type or *KRAS* mutant CRC (Fig. S1B).

Previously, *TFF2* expression was demonstrated to be associated with *BRAF* V600E mutation in CRC [20]. As such, the present investigation focused on the role and mechanism of *HPSE* in *BRAF* V600E-mutant CRC. Multivariate analyses of all parameters unbalanced between *BRAF* V600E-mutant and wild-type groups demonstrated a significant and independent correlation of *BRAF* V600E mutation with *HPSE* expression in TCGA and GSE39582 datasets (Fig. 1B). Notably, a significant upregulation of *HPSE* expression was reported in *BRAF* V600E-mutant CRC, independent of MMR status (Fig. 1C, Fig. S2). Finally, we examined *HPSE* mRNA levels in 44 CRC samples (Clinical characteristics are shown in Table S2) from Zhongshan hospital Fudan University and the analysis revealed a notably higher *HPSE* expression in *BRAF* V600E-mutant CRC compared to *BRAF* wide-type (Fig. 1D).

### *HPSE* expression is associated with the prognosis of *BRAF* V600E-mutant CRC

**Fig. 2:**
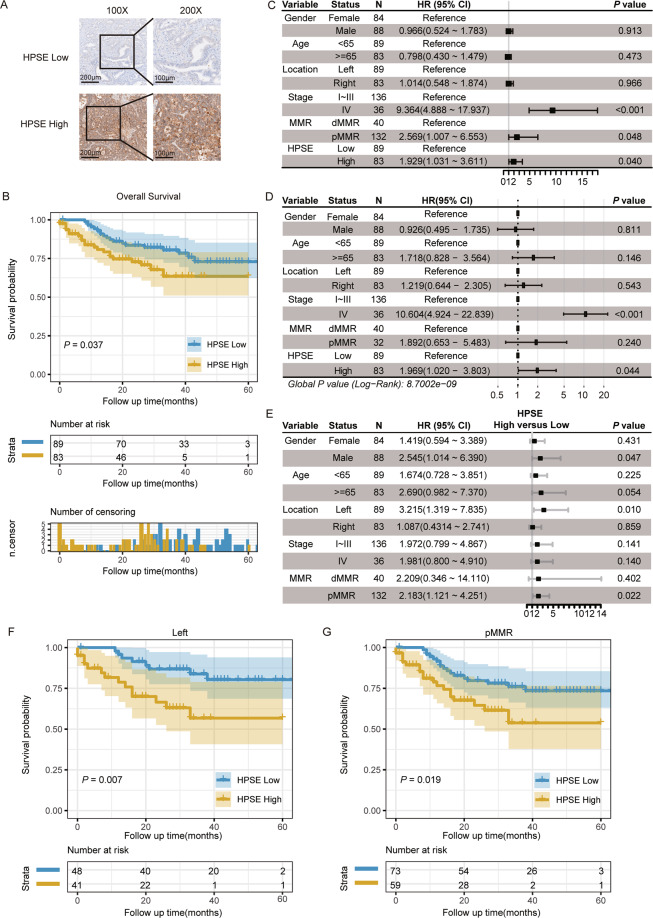
High HPSE expression level is associated with poor prognosis of *BRAF* V600E-mutant CRC patients. **A** Representative images of immunohistochemical staining for HPSE. **B** Kaplan–Meier survival curves in HPSE high and low groups of patients with *BRAF* V600E-mutant CRC. A log-rank test was used to calculate the *P*-value. Shaded regions around the curves indicate 95% confidence intervals. The number of patients at risk and censoring are presented below the survival curve. **C** Results of univariate Cox proportional hazard model presented as Hazard ratios (blocks in the center) and 95% confidence intervals (Whiskers of error bars). **D** Results of multivariate Cox proportional hazard model presented as Hazard ratios (blocks in the center) and 95% confidence intervals (Whiskers of error bars). **E** Results of survival analysis in different subgroups. Hazard ratios (blocks in the center) and 95% confidence intervals (Whiskers of error bars) were determined by univariate Cox regression. The reference level in each subgroup was HPSE low. Kaplan–Meier survival curves in the (**F**) left location subgroup and (**G**) pMMR subgroup. A log-rank test was used to determine *P*-value. Shaded regions around the curves indicate 95% confidence intervals. The number of patients at risk are presented below the survival curve. R survival package, survminer package, and forest package were used for survival analysis. HR hazard ratios; CI confidence intervals.

We collected samples of *BRAF* V600E-mutant CRC patients from 7825 patients diagnosed with colorectal adenocarcinoma, who underwent surgery and genetic analysis of *BRAF* at Zhongshan Hospital Fudan University between June 2015 and December 2018. A total of 172 *BRAF* V600E-mutant CRC patients were retrospectively analyzed to explore the prognostic role of *HPSE* in *BRAF* V600E-mutant CRC. Clinicopathological characteristics of the 172 patients are presented in Table S3. IHC staining evaluation of *HPSE* expression revealed that 158 samples were HPSE positive. Patients were assigned into HPSE high (*N* = 83) and HPSE low (*N* = 89) groups based on the median expression level of *HPSE*. Of note, the HPSE high group exhibited poor differentiation (Table S3 and Fig. 2A). Kaplan–Meier survival analysis demonstrated significantly lower overall survival of the HPSE high group compared to that of the HPSE low group (Fig. 2B). Stage IV, proficient mismatch repair (pMMR), and high *HPSE* expression were markedly associated with inferior survival in the univariable Cox proportional hazards model (Fig. 2C). Furthermore, multivariable Cox model analysis provided evidence that *HPSE* expression is a prognostic factor independent of clinicopathological parameters, including gender, age, location, stage, and MMR status (Fig. 2D).

Moreover, exploration of the prognostic significance of *HPSE* expression in different subgroups revealed that a significant prognostic value was preserved in male, left location, and pMMR subgroups (Fig. 2E). Intriguingly, a more noticeable prognostic value was reported in the left location (Fig. 2F) and pMMR subgroups (Fig. 2G) compared to that of the overall population. In stage I~III or IV subgroup, survival curves also revealed differences between HPSE high and low groups (Fig. S3). The median overall survival time of patients with stage IV CRC was 21 months in the HPSE low group, whereas stage IV CRC patients showed a median overall survival of 8 months in the HPSE high group (Fig. S3B). These data demonstrate a robust prognostic value of *HPSE* expression in *BRAF* V600E-mutant CRC patients.

### *HPSE* silencing inhibits the proliferation of *BRAF* V600E-mutant CRC cells in vitro and in vivo

**Fig. 3:**
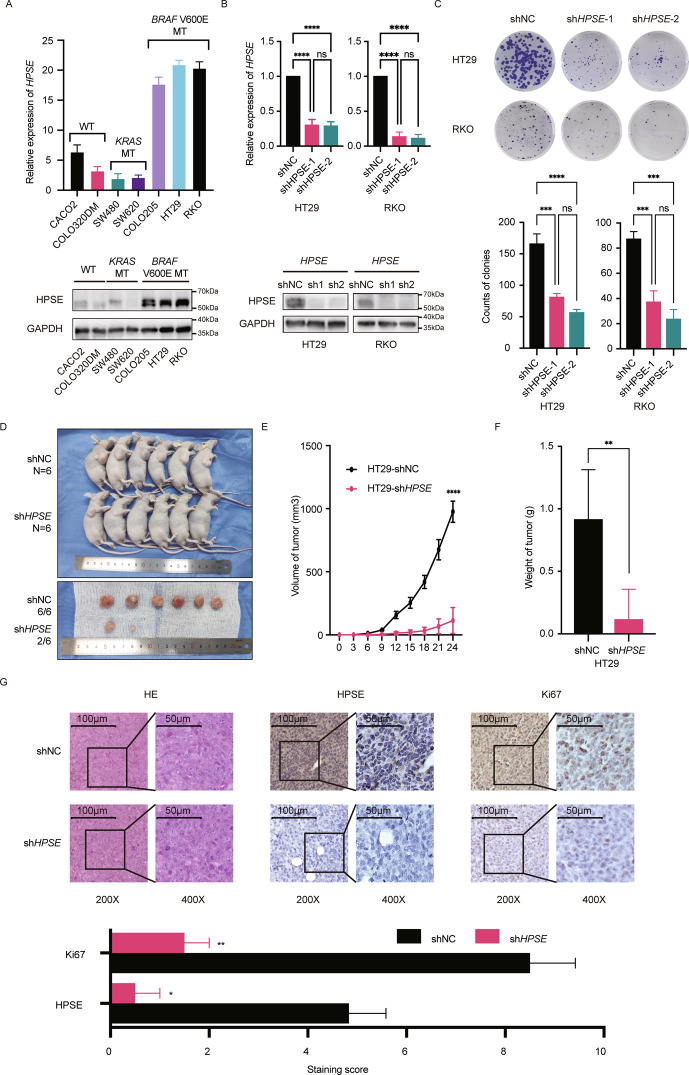
Silencing *HPSE* inhibits proliferation of *BRAF* V600E-mutant CRC cells in vitro and in vivo. **A** Baseline relative expression level of *HPSE* in different CRC cell lines at mRNA level (top) and protein level (bottom). The same trend was observed in three independent repeated experiments. Representative results are shown. WT: *KRAS/BRAF* wild type; *KRAS* MT: *KRAS* mutant type; *BRAF* V600E MT: *BRAF* V600E mutant type. **B** Verification of knockdown of *HPSE* expression in HT29 and RKO at mRNA level (top) and protein level (bottom). The same trend was observed in three independent repeated experiments. Representative results are shown. Data are presented as mean ± standard deviation. NC negative control. **C** Representative images and statistical analysis of colony formation assay. Cells were seeded in six-well plates at 500 cells per well and cultured for 14 days. The same trend was observed in three independent repeated experiments. Data are presented as the mean ± standard deviation. **D** Subcutaneous xenograft models and tumors isolated on day 24. Each group contained six mice. Subcutaneous tumor formation rates of the two groups were 100% (6/6) in the control group and 33.3% (2/6) in the *HPSE* silencing group. **E** Growth curves of subcutaneous tumors in nude mice. Points and error bars represent mean ± standard errors. **F** Weight of subcutaneous tumors isolated from models on day 24. Data are presented as the mean ± standard deviation. **G** HE staining and IHC staining of HPSE and Ki67 in subcutaneous tumor tissues. Representative images and semi-quantification analysis are shown. Data are presented as the mean ± standard deviation. The student’s *t*-test was used to determine the *P*-value in two-group comparisons. One-way ANOVA analysis and Tukey’s test were used for multiple comparisons. ****P < 0.0001, ***P < 0.001, **P < 0.01, *P < 0.05, ns *P* > 0.05.

Cell-based and animal model experiments were performed to explore the function of *HPSE* in *BRAF* V600E-mutant CRC cells. Seven CRC cell lines with different gene types were used to explore the baseline expression level of *HPSE*. Three *BRAF* V600E-mutant CRC cell lines showed significantly higher *HPSE* expression at both transcriptional and translational levels compared to wild-type cells (Fig. 3A). Two *BRAF* V600E-mutant CRC cell lines, HT29 and RKO, were selected to stably silence *HPSE* expression using lentiviral shRNA. HPSE expression and its enzymatic activity were significantly inhibited in cells with *HPSE* silencing (Fig. 3B and Fig. S4A, B). Colony formation assays were subsequently performed to explore cell proliferation rate. Results showed that *HPSE* silencing significantly suppressed the proliferation of the two *BRAF* V600E-mutant CRC cell lines (Fig. 3C). Furthermore, subcutaneous xenograft models were used to investigate the effect of *HPSE* repression on tumor growth. HT29 cells with silenced *HPSE* (HT29-sh*HPSE*) and the control (HT29-shNC) were subcutaneously administered into nude mice and the tumor size was recorded until any of the mice attained a tumor volume of 1000 mm^3^. Analysis showed that, of the six mice in the HT29-sh*HPSE* group, two had significantly increased tumor sizes, whereas 100% measurable tumors were formed in the HT29-shNC group (Fig. 3D). Enzyme assay was performed to confirm the suppressed enzymatic activity (Fig. S4C). Tumor growth curves showed a significant decrease in growth rate in *HPSE* silenced tumors (Fig. 3E). Tumors resected from the HT29-sh*HPSE* group were much smaller, with a significantly lower average weight (Fig. 3F) and lower Ki67 expression level compared to those resected from the HT29-shNC group (Fig. 3G). These data demonstrate a crucial role for *HPSE* in cell proliferation of *BRAF* V600E-mutant CRC in vitro and in vivo.

### *HPSE* silencing induces cell cycle arrest of *BRAF* V600E-mutant CRC by downregulating Cyclin E2 expression

**Fig. 4:**
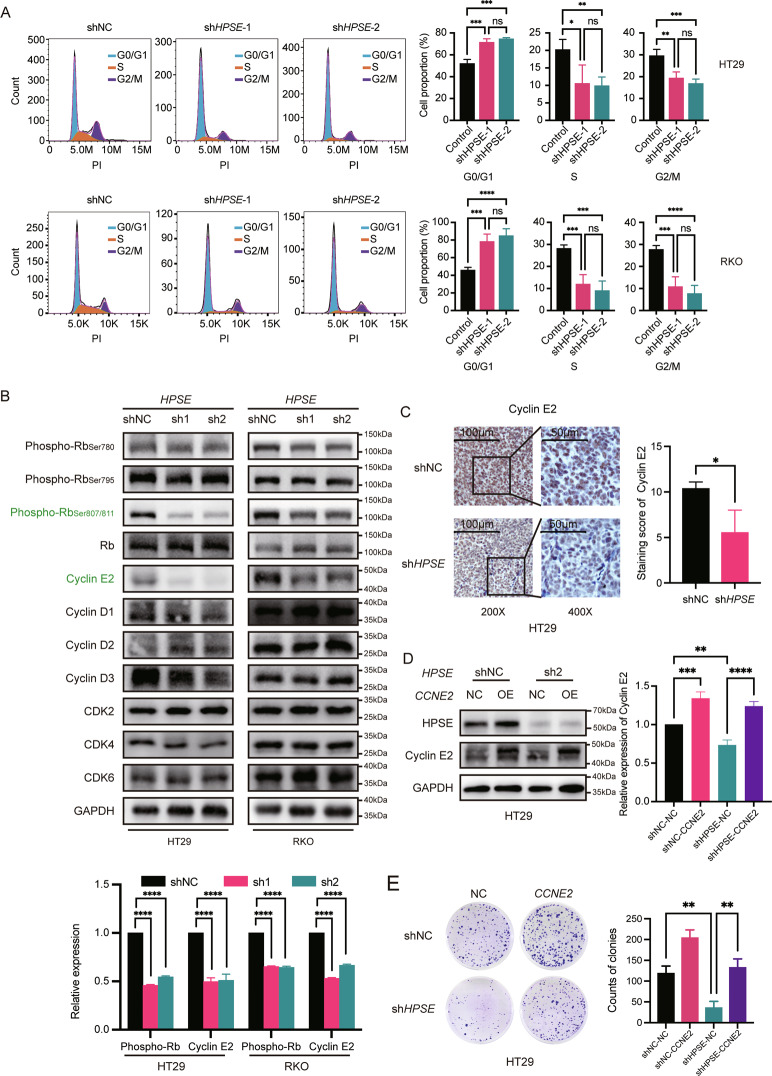
Silencing *HPSE* arrests cell cycle of *BRAF* V600E-mutant CRC cells by downregulating Cyclin E2 expression. **A** Cell cycle distributions and statistical analysis of HT29 and RKO with or without *HPSE* silencing. The same trend was observed in three independent repeated experiments. Representative images and statistical analysis of cell cycle distribution are shown. Data are presented as the mean ± standard deviation. **B** Expressions of critical cell cycle-related proteins in *BRAF* V600E-mutant CRC cells with or without *HPSE* silencing. The same trend was observed in three independent repeated experiments. Green color represents downregulated proteins. Representative results and semi-quantification analysis are shown. Data are presented as the mean ± standard deviation. **C** IHC staining of Cyclin E2 in subcutaneous tumor tissues. Representative results and semi-quantification analysis are shown. Data are presented as the mean ± standard deviation. **D** Verification of *CCNE2* overexpression in *HPSE-*knockdown HT29 at the protein level. The same trend was observed in three independent repeated experiments. Representative results and semi-quantification analysis are shown. Data are presented as the mean ± standard deviation. NC negative control. OE overexpression of *CCNE2*. **E** Representative images and statistical analysis of colony formation assay. Cells were seeded in six-well plates at 1000 cells per well and cultured for 10 days. The same trend was observed in three independent repeated experiments. Data are presented as the mean ± standard deviation. The student’s *t*-test was used to determine the *P*-value in two-group comparisons. One-way ANOVA analysis and Tukey’s test were used for multiple comparisons. ****P < 0.0001, ***P < 0.001, **P < 0.01, *P < 0.05, ns *P* > 0.05.

Further analyses were performed to explore the mechanism underlying the *HPSE* effect on cell proliferation. Cell cycle distribution was analyzed via flow cytometry with PI staining. *BRAF* V600E-mutant CRC cells with silenced *HPSE* were significantly arrested at G0/G1 phase, whereas cell proportions of S and G2/M phases were much smaller compared to the proportions of the control group (Fig. 4A). Western blot detection of the protein levels of key molecules involved in cell cycle progression showed that the levels of phosphorylated Rb and Cyclin E2 were decreased significantly in *HPSE*-silenced HT29 and RKO cells than the levels in the control group (Fig. 4B). In addition, the expression of Cyclin E2 was downregulated in xenograft tissues from the HT29-sh*HPSE* group compared to that of the control group (Fig. 4C). Notably, overexpression of *CCNE2* (encoding Cyclin E2) in *HPSE*-silenced cells (Fig. 4D) restored the cell proliferation ability (Fig. 4E).

### *HPSE* silencing inhibits the proliferation of *BRAF* V600E-mutant CRC cells by regulating the AKT/p27Kip1 pathway

**Fig. 5:**
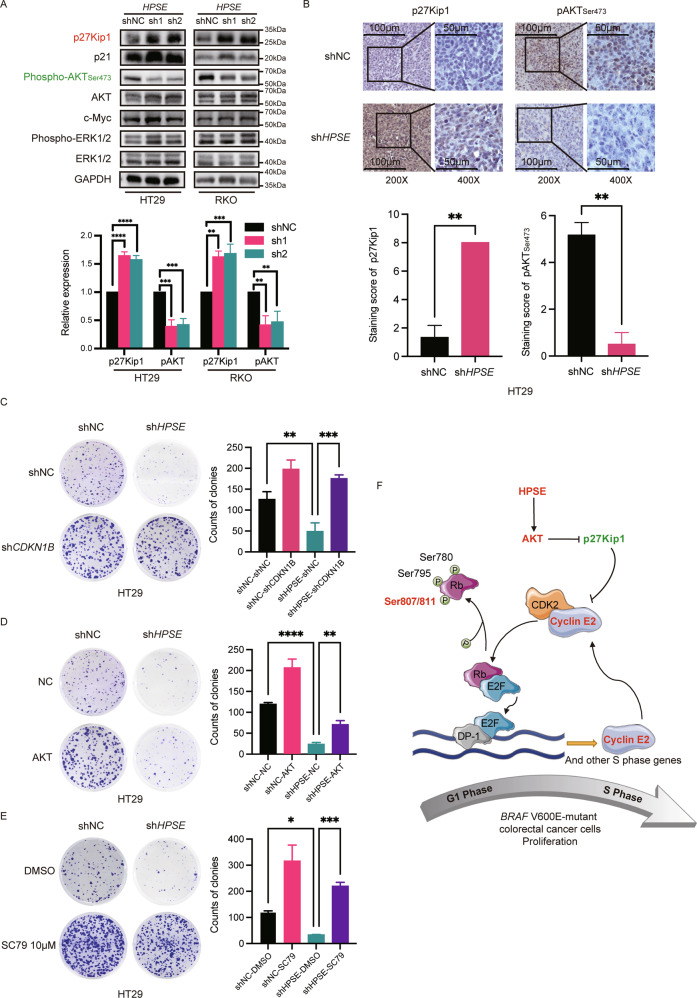
Silencing *HPSE* suppresses cell proliferation of *BRAF* V600E-mutant CRC cells through AKT/p27Kip1 pathway. **A** Expression levels of cell proliferation-related signaling proteins in *BRAF* V600E-mutant CRC cells with or without *HPSE* silencing. Red color represents upregulated proteins. Green represents downregulated proteins. The same trend was observed in three independent repeated experiments. Representative images and semi-quantification analysis are shown. Data are presented as the mean ± standard deviation. **B** IHC staining of p27Kip1 and phospho-AKT_ser473_ in subcutaneous tumor tissues. Representative images and semi-quantification analysis are shown. Data are presented as the mean ± standard deviation. **C**–**E** Representative images and statistical analysis of colony formation assay. Cells were seeded in six-well plates at 1000 cells per well and cultured for 10 days. In the group treated with SC79, SC79 (S7863, Selleckchem, Shanghai, China) was dissolved in dimethyl sulfoxide (DMSO, Sigma) and diluted to 10 µM with the complete medium before use. Cells were incubated with DMSO/SC79 (10 µM) for 7 days. The same trend was observed in three independent repeated experiments. Data are presented as the mean ± standard deviation. **F** Proposed model for the mechanism of HPSE function on cell proliferation in *BRAF* V600E-mutant CRC cells. Red color represents upregulated or activated proteins. Green represents downregulated or inactivated proteins. The student’s t-test was used to determine the *P*-value in two-group comparisons. One-way ANOVA analysis and Tukey’s test were used for multiple comparisons. ****P < 0.0001, ***P < 0.001, **P < 0.01, *P < 0.05, ns *P* > 0.05.

Analysis of upstream cell cycle regulators revealed significant upregulation of p27Kip1 and marked downregulation of AKT phosphorylation following HPSE repression (Fig. 5A), which was validated in mice xenografts (Fig. 5B). To further confirm that HPSE specifically regulated AKT phosphorylation and p27Kip1 levels, phosphorylation of c-Myc and ERK, which play important roles in cell proliferation were explored. Results showed no significant changes in phosphorylation level of them (Fig. 5A). Besides, *CDKN1B* (encoding p27Kip1) knockdown significantly restored the cell proliferation capacity (Fig. 5C) and upregulated expression of Cyclin E2 (Fig. S5A, B) which was impaired by HPSE knockdown. AKT overexpression in *HPSE*-silenced cells only slightly rescued the phosphorylation level of AKT (Fig. S5C, D) and the phenotype (Fig. 5D), whereas the upregulation of p27Kip1 and the inhibition of Cyclin E2 were remained (Fig. S5C, D). Furthermore, when cell models were treated with SC79 (AKT phosphorylation activator), the results showed that the effects of *HPSE*-silencing were completely rescued (Fig. 5E, Fig. S5E, F). The findings provide evidence that HPSE promotes cell proliferation by regulating cell cycle progression via the AKT/p27Kip1/Cyclin E2 axis in *BRAF* V600E-mutant colorectal cancer (Fig. 5F).

## Discussion

Colorectal cancer carrying *BRAF* V600E mutation intrigued many researchers due to its poor prognosis. *BRAF* V600E-mutant population has a unique gene expression pattern that is lacking in *BRAF* wild-type CRC subjects. Researchers have developed a 32-gene signature based on analysis of DEGs to identify *BRAF* V600E-mutant patients and predict their prognosis [10]. In the present study, by analyzing the transcriptome sequencing data of two public datasets, *HPSE* was revealed to be significantly highly expressed in *BRAF* V600E-mutant CRC compared to the wild type CRC. Mounting evidence indicates that HPSE protein is a key player in cancer progression [15]. In our study, multiple linear regression analysis and verification using clinical samples showed a significant correlation between *BRAF* V600E mutation and high *HPSE* expression. Previous evidence indicates that *BRAF* V600E mutation causes continuous abnormal activation of the MAPK pathway, and a series of downstream effects on key cell processes, including cell proliferation, differentiation, and apoptosis [3, 8]. CpG island methylation phenotype is also associated with *BRAF* V600E mutation; however, the mechanism has not been elucidated [21]. In the context of these complex molecular biological characteristics, the detailed mechanism for increased expression of *HPSE* in *BRAF* V600E-mutant CRC warrants further exploration.

Moreover, whether high *HPSE* expression contributes to the poor prognosis of *BRAF* V600E-mutant CRC patients remains to be determined. Although researchers have explored the association of *HPSE* expression and survival outcome of patients with CRC, these studies used small sample sizes (130 cases [22] and 54 cases [23], respectively) and did not explore the correlation with *BRAF* status. In the present study, the prognostic value of *HPSE* expression was confirmed at the protein level using a large retrospective cohort of 172 *BRAF* V600E-mutant patients. Results revealed *HPSE* expression to be a prognostic factor of *BRAF* V600E-mutant CRC, independent of gender, age, primary location, tumor stage, and MMR status.

In addition, subgroup analysis showed that the prognostic value of *HPSE* expression was more significant in patients with left-side primary tumor or pMMR. Previously, researchers have demonstrated significant differences in clinical features and molecular features between left and right CRC, which are potentially correlated with the different prognostic effects of *HPSE* expression in these two populations [24, 25]. Currently, MMR status is one essential molecular classification parameter of CRC in the immunotherapy era. Deficient mismatch repair (dMMR) patients benefit more from immunotherapy and present better survival regardless of *BRAF* mutation compared to patients with pMMR patients [26]. Nevertheless, effective management strategies are needed to be recommended for pMMR patients, particularly those carrying *BRAF* V600E mutation. Our recently research suggested that pMMR mCRC with high HPSE expression might respond to immune checkpoint inhibitors [27]. The significant prognostic value of *HPSE* in *BRAF* V600E-mutant CRC provides a valuable guide for the prognosis prediction and identification of effective therapeutic strategy for this population.

HPSE protein is a multifunctional cancer-promoting molecule and through its enzymatic activity, it hydrolyzes heparan sulfate to trigger remodeling the extracellular matrix, promote invasion and metastasis, and induce changes in the tumor microenvironment. HPSE protein also plays a role in signal transduction and regulation of transcription of genes independent of its enzymatic activity [12, 15]. In this view, we investigated the function of *HPSE* in *BRAF* V600E-mutant CRC cells. Of note, *HPSE* silencing significantly inhibited the proliferation of *BRAF* V600E-mutant CRC cells. Xenograft models further confirmed the role of *HPSE* in tumor growth. Along with the decrease in Rb phosphorylation level and Cyclin E2 protein expression, the cell cycle of *BRAF* V600E-mutant CRC cells was significantly blocked in G0/G1 phase in *HPSE*-silenced cells. At the same time, *HPSE* silencing decreased the phosphorylation of AKT and increased p27Kip1 expression. Accordingly, we speculated that high *HPSE* expression in *BRAF* V600E-mutant CRC cells enhances cell proliferation by promoting cell cycle progression through AKT signaling pathway activation and inhibition of p27Kip1 protein expression, which was supported strongly by rescue experiments. These findings demonstrate that HPSE is a promising therapeutic target for *BRAF* V600E-mutant CRC.

It has been reported that the addition of exogenous HPSE precursors in endothelial cells activates AKT through its non-enzymatic functions [28]. The carboxy-terminal domain of HPSE protein is implicated in the mediation of AKT signaling transduction independent of its enzymatic activity [29]. HPSE was previously reported to promote tumor growth and metastasis in breast cancer by modulating phosphorylation of AKT, STAT5, and SRC [30]. Supported by previous findings, the present study revealed that HPSE modulates the phosphorylation level of AKT in *BRAF* V600E mutant CRC. Cyclin-E family, Rb protein, and its phosphorylation along with p27Kip1 protein have all been shown to play essential roles in G1/S phase transition [31]. In particular, Cyclin-E activates CDK2 and initiates the transition from G1 phase to S phase, and its expression is dependent on E2F transcription factors [32]; Rb protein is a tumor suppressor that binds to transcription factor E2F to make it non-transactivated in G0/G1 phase [33]; Cyclin D-CDK4/6 and Cyclin E-CDK2 complexes phosphorylate Rb protein, which then to dissociate from E2F, and consequently promote expression of Cyclin E and other S phase-related genes [34]. p27Kip1 is a member of the CDK inhibitor protein family and has been shown to inhibit the G1/S phase transition of the cell cycle by disrupting the function of Cyclin E-CDK2 [31]. The AKT signaling pathway regulates p27Kip1 protein at transcription and post-transcription levels [35]. Findings of the present study demonstrated that HPSE modulates the AKT/p27Kip1 pathway in *BRAF* V600E-mutant CRC, playing a part in the regulation of G1/S phase transition of the cell cycle, and consequently influences cell proliferation. However, silencing *HPSE* expression by shRNA decreased the protein level and also inhibited the enzymatic activity in our study. It remains uncertain that whether HPSE contributes via its non-enzymatic activity. A full picture of the mechanism warrants further exploration.

In conclusion, the potential contribution of *HPSE* to the poor prognosis of *BRAF* V600E-mutant CRC has been established through bioinformatic analyses. The role of HPSE as a prognostic biomarker was explored in the retrospective cohort. Through in vitro and in vivo experiments, this work revealed the function of *HPSE* in tumor development by regulating the cell cycle via the AKT/p27Kip1 pathway. The findings strongly demonstrate that HPSE holds great promise as a therapeutic target for *BRAF* V600E-mutant CRC.

## Materials/subjects and methods

### Analysis of DEGs

All the procedures of DEGs analysis were performed in R (version 3.6.3) [36]. Data on CRC patients were retrieved from The Cancer Genome Atlas (TCGA) using the TCGAbiolinks package [37]. The GSE39582 dataset [38] was retrieved from the Gene Expression Omnibus (GEO) database. DESeq2 [39] and limma [40] packages were employed to explore DEGs between patients with and without *BRAF* V600E mutation in TCGA and GSE39582 datasets, respectively. Significantly DEGs with log2 expression fold change (Log2 FC) > 1 or <−1 and adjusted *P* < 0.05 were sorted based on the adjusted *P*-value. The common genes from the top-ten upregulated, and top-ten downregulated genes were selected from the two datasets. Multiple linear regression analysis was conducted using the lm function in the R stats package.

### Patient samples

Frozen tumor tissues were collected from 44 patients with metastatic CRC who underwent surgery at Zhongshan Hospital Fudan University. There were 7825 patients undergoing surgery for primary tumor of CRC and genetic analysis of *BRAF* at Zhongshan Hospital Fudan University between June 2015 and December 2018. Among them, formalin-fixed paraffin-embedded (FFPE) tumor tissues of 172 patients with *BRAF* V600E-mutant CRC were retrospectively collected according to the following inclusion criteria: (1) Patients diagnosed with colorectal adenocarcinoma at Zhongshan Hospital Fudan University between June 2015 and December 2018. (2) Patients who underwent surgery for primary tumor of CRC, and had not received systemic or local anti-tumor treatment before surgery. (3) Patients with *BRAF* V600E mutation of the primary tumor tissue confirmed via genetic analysis. The exclusion criteria were as follows: (1) Patients with no available FFEP tumor tissue of primary focus. (2) Patients with CRC and other concomitant primary malignant tumors or diagnosed with a hereditary tumor.

Clinical and pathological information was retrieved from the electronic medical records. Postoperative pathological staging for each patient was based on the American Joint Committee on Cancer (AJCC) cancer staging guidelines, 8th edition. The median follow-up time for the retrospective cohort was 32 months. Overall survival (OS) was defined as the time from surgery to death for any reason or the time of the last follow-up.

### Immunohistochemistry analysis

Immunohistochemistry (IHC) staining of FFEP slides from patients and xenografts was conducted using an automated system (BenchMark XT, Roche). HPSE antibody (ab85543, Abcam, Cambridge, UK) and p27Kip1 antibody (25614-1-AP, Proteintech, Wuhan, China) at a concentration of 1:100, Ki67 antibody (GB111499, Servicebio, Wuhan, China) 1:200, Cyclin E2 (11935-1-AP, Proteintech) 1:500, Phospho-Akt (Ser473) antibody (4060, CST, MA, USA) 1:50 were used for immunohistochemistry analysis. Two independent pathologists blinded to the study evaluated the staining score of each clinical sample based on the staining extent (0, no staining; 1, weak staining; 2, moderate staining; 3, strong staining) and intensity (0, 0–5%; 1, 6% ~ 25%; 2, 26% ~ 50%; 3, 51% ~ 75%; 4, 75% ~ 100%) of tumor cells. HPSE positive represented more than 5% staining intensity of the tumor cells. Samples with staining extent and intensity scores greater than 1 were denoted as HPSE high, whereas those with scores less than 1 were denoted as HPSE low.

### Cell culture

Human embryonic kidney cell line, 293 T and human CRC cell lines, CACO2, COLO320DM, SW480, SW620, COLO205, HT29, and RKO were purchased from the National Collection of Authenticated Cell Cultures (Shanghai, China). CACO2 and COLO320DM were *KRAS/BRAF* wild-type cell lines. SW480 and SW620 were *KRAS* mutant and *BRAF* wild-type cell lines. COLO205, HT29, and RKO were *BRAF* V600E-mutant cell lines. 293 T, HT29, and RKO cells were cultured in DMEM/high glucose medium (SH30022.01B, HyClone, Logan, UT, USA) supplemented with 10% FBS (16140071, Gibco, Paisley, UK) and 1% Pen/Strep (16140071, Gibco, Paisley, UK). CACO2 cells were cultured in DMEM/high glucose medium (HyClone) supplemented with 20% FBS (Gibco) and 1% Pen/Strep (Gibco). COLO320DM and COLO205 cells were cultured in RMPI 1640 (HyClone) supplemented with 10% FBS (Gibco) and 1% Pen/Strep (Gibco). 293 T, HT29, RKO, CACO2, COLO320DM, and COLO205 cells were cultured at 37 °C under 5% CO_2_. SW480 and SW620 cells were cultured in Leibovitz’s L15 medium (11415064, Gibco, Paisley, UK) supplemented with 10% FBS (Gibco) and 1% Pen/Strep (Gibco) at 37 °C without CO_2_. Short tandem repeat profiling and mycoplasma test were performed for all cell lines to confirm the cell identity and ensure the cells were free from contamination.

### Construction of stable transfectants

Plasmids for *HPSE* silencing were purchased from Genechem (Shanghai, China). Plasmids for *CCNE2* overexpression, *CDKN1B* silencing, and *AKT1* overexpression were purchased from Genomeditech (Shanghai, China). Lentivirus was generated using 293 T cells and transduced into cells. Stably transfected cells were selected using puromycin and blasticidin. shRNA targeting sequences for *HPSE* and *CDKN1B* were as follows:

sh*HPSE*-1: 5’-TTCCTGAAGGCTGGTGGAGAA-3’,

sh*HPSE*-2: 5’-CTCCGAGAACACTACCAGAAA-3’,

sh*CDKN1B*: 5’-GCAACCGACGATTCTTCTACT-3’.

### Colony formation assay

Cells were seeded in six-well plates at 500 cells per well and cultured for 14 days or 1000 cells for 10 days. Colonies were fixed with 4% paraformaldehyde for 15 min and stained with 0.1% crystal violet for 30 min. Colonies were then scanned as images and quantified using ImageJ software (Version 1.53a) [41].

### Subcutaneous xenograft models

Six-week-old male *BALB/*c nude mice were purchased from SLAC Laboratory (Shanghai, China). HT29-sh*HPSE* cells and control cells were resuspended in PBS at a density of 1 × 10^7 cells/ml. Cell suspensions (100 μl) were injected subcutaneously into the left axillary area of the nude mice. Twelve mice were randomized into two groups. Mice were monitored daily, and the size of palpable tumors was determined every three days. Tumor volumes were calculated using the formula: π/6 x Length x Width^2. All mice were euthanized when the volume of any of the model mice reached a volume of 1000 mm^3. Tumors were weighed and fixed in 4% paraformaldehyde. FFEP slides of the tumors were prepared for hematoxylin-eosin (HE) and IHC staining. Investigators were blind to the status of the particular mouse during the measurement, dissection, weighing, and photographing of the tumors.

### Cell cycle assay

Cells for cell cycle examination were harvested, fixed, and stained with propidium iodide (CCS012, LinkTech, Shanghai, China) according to the manufacturer’s instructions. Flow cytometry was used to explore cell cycle distribution. Results were analyzed using Flowjo software (Version 10.6.2. Ashland, OR: Becton, Dickinson, and Company; 2019).

### Heparanase enzyme assay

Heparan degrading enzyme assay kit (MK412, Takara, Shiga, Japan) was used to measure the heparanase enzymatic activity of cells and xenograft tissues following the manufacturer’s instruction.

### RNA extraction and reverse transcription-quantitative polymerase chain reaction (RT-qPCR)

Total RNA was extracted from frozen CRC tissues and cells using Total RNA Kit I (R6834-02, Omega Bio-Tek, GA, USA) and RNase-Free DNase Set (E1091-02, Omega Bio-Tek, GA, USA) following the manufacturer’s instructions. RNA was reverse transcribed using PrimeScript RT Master Mix (Perfect Real Time) kit (RR036A, Takara, Shiga, Japan). TB Green Premix Ex Taq (Tli RNaseH Plus) kit (RR420A, Takara, Shiga, Japan) was used to perform quantitative PCR. Primers for *HPSE* and *GAPDH* were synthesized by Sangon (Shanghai, China) and are listed in Table S4. The 2 (-Delta Delta Ct) method [42] was used for relative quantitative analysis of the expression data using *GAPDH* as an internal reference gene.

### Western blot (WB) analysis

Cells were lysed in SDS lysis buffer (P0013G, Beyotime Biotechnology, Shanghai, China) supplemented with proteinase/phosphatase inhibitor mixture (P1046, Beyotime Biotechnology, Shanghai, China) for protein extraction. BCA kit (P0012, Beyotime Biotechnology, Shanghai, China) was used to determine the total protein concentration. Equal amounts of protein were electrophoresed on 10% SDS–PAGE gels and transferred to nitrocellulose membranes. Membranes were blocked with 3% BSA solution and incubated with diluted primary antibodies overnight at 4 °C. Further, membranes were washed and incubated with appropriate secondary antibodies for 1 h at room temperature. Protein bands were visualized by an enhanced chemiluminescent method. Antibodies used for WB in this study were as follows: HPSE antibody (ab254254, Abcam), Cell cycle regulation antibody sampler kit (9932, CST), Rb antibody sampler kit (9969, CST), Cyclin E2 antibody (4132, CST), Cyclin D2 antibody (3741, CST), Cyclin D3 antibody (2936, CST), Phospho-Erk1/2 (Thr202/Tyr204) antibody (4370, CST), Erk1/2 antibody (4695, CST), Phospho-Akt (Ser473) antibody (4060, CST), AKT antibody (4691, CST), GAPDH antibody (5174, CST), anti-rabbit IgG, HRP-linked antibody (7074, CST), anti-mouse IgG and HRP-linked antibody (7076, CST). Semi-quantitative analysis of bands was performed using ImageJ software (Version 1.53a) [41].

### Statistical analysis

R packages and statistical methods used for each analysis are described in Figure legends. For all experiments, there were at least three independent replicates. Statistical tests were two-sided if applicable. *P* < 0.05 was considered statistically significant. All statistical analyses and data plotting were performed in RStudio (Version 1.2.1335, R version 3.6.3) [36] and GraphPad Prism software (Version 9.0.0 for Mac OS X, GraphPad Software, San Diego, California USA, www.graphpad.com).

## Supplementary information

Table S1, 2, 4 and Figure S1–5

Table S3

## Data Availability

The datasets used and/or analyzed during the current study are available from the corresponding author on reasonable request.

## References

[1] Cancer Genome Atlas N. (2012). Comprehensive molecular characterization of human colon and rectal cancer. Nature.

[2] Sanz-Garcia E, Argiles G, Elez E, Tabernero J (2017). BRAF mutant colorectal cancer: prognosis, treatment, and new perspectives. Ann Oncol.

[3] Yao Z, Yaeger R, Rodrik-Outmezguine VS, Tao A, Torres NM, Chang MT (2017). Tumours with class 3 BRAF mutants are sensitive to the inhibition of activated RAS. Nature.

[4] Seligmann JF, Fisher D, Smith CG, Richman SD, Elliott F, Brown S (2017). Investigating the poor outcomes of BRAF-mutant advanced colorectal cancer: analysis from 2530 patients in randomised clinical trials. Ann Oncol.

[5] Roth AD, Tejpar S, Delorenzi M, Yan P, Fiocca R, Klingbiel D (2010). Prognostic role of KRAS and BRAF in stage II and III resected colon cancer: results of the translational study on the PETACC-3, EORTC 40993, SAKK 60-00 trial. J Clin Oncol.

[6] Bläker H, Alwers E, Arnold A, Herpel E, Tagscherer KE, Roth W (2019). The Association Between Mutations in BRAF and Colorectal Cancer-Specific Survival Depends on Microsatellite Status and Tumor Stage. Clin Gastroenterol Hepatol.

[7] Wang J, Shen J, Huang C, Cao M, Shen L (2019). Clinicopathological Significance of BRAF(V600E) Mutation in Colorectal Cancer: An Updated Meta-Analysis. J Cancer.

[8] Caputo F, Santini C, Bardasi C, Cerma K, Casadei-Gardini A, Spallanzani A *et al*. BRAF-Mutated Colorectal Cancer: Clinical and Molecular Insights. *International journal of molecular sciences* 2019; 20. 10.3390/ijms20215369 PMC6861966 31661924

[9] Chen G, Gao C, Gao X, Zhang DH, Kuan SF, Burns TF (2018). Wnt/β-Catenin Pathway Activation Mediates Adaptive Resistance to BRAF Inhibition in Colorectal Cancer. Mol Cancer Ther.

[10] Popovici V, Budinska E, Tejpar S, Weinrich S, Estrella H, Hodgson G (2012). Identification of a poor-prognosis BRAF-mutant-like population of patients with colon cancer. J Clin Oncol.

[11] Vecchione L, Gambino V, Raaijmakers J, Schlicker A, Fumagalli A, Russo M (2016). A Vulnerability of a Subset of Colon Cancers with Potential Clinical Utility. Cell.

[12] Vlodavsky I, Singh P, Boyango I, Gutter-Kapon L, Elkin M, Sanderson RD (2016). Heparanase: From basic research to therapeutic applications in cancer and inflammation. Drug Resist Updat.

[13] Vlodavsky I, Gross-Cohen M, Weissmann M, Ilan N, Sanderson RD (2018). Opposing Functions of Heparanase-1 and Heparanase-2 in Cancer Progression. Trends Biochem Sci.

[14] Masola V, Bellin G, Gambaro G, Onisto M Heparanase: A Multitasking Protein Involved in Extracellular Matrix (ECM) Remodeling and Intracellular Events. *Cells* 2018; 7. 10.3390/cells7120236 PMC6316874 30487472

[15] Masola V, Zaza G, Gambaro G, Franchi M, Onisto M (2020). Role of heparanase in tumor progression: Molecular aspects and therapeutic options. Semin Cancer Biol.

[16] Mohan CD, Hari S, Preetham HD, Rangappa S, Barash U, Ilan N (2019). Targeting Heparanase in Cancer: Inhibition by Synthetic, Chemically Modified, and Natural Compounds. iScience.

[17] Liu X, Zhou ZH, Li W, Zhang SK, Li J, Zhou MJ (2019). Heparanase Promotes Tumor Growth and Liver Metastasis of Colorectal Cancer Cells by Activating the p38/MMP1 Axis. Front Oncol.

[18] Katakam SK, Pelucchi P, Cocola C, Reinbold R, Vlodavsky I, Greve B (2020). Syndecan-1-Dependent Regulation of Heparanase Affects Invasiveness, Stem Cell Properties, and Therapeutic Resistance of Caco2 Colon. Cancer Cells. Front Oncol.

[19] Rao G, Liu D, Xing M, Tauler J, Prinz RA, Xu X (2010). Induction of heparanase-1 expression by mutant B-Raf kinase: role of GA binding protein in heparanase-1 promoter activation. Neoplasia.

[20] Gala MK, Austin T, Ogino S, Chan AT (2015). TFF2-CXCR4 Axis Is Associated with BRAF V600E Colon Cancer. Cancer Prev Res (Philos).

[21] Tao Y, Kang B, Petkovich DA, Bhandari YR, In J, Stein-O’Brien G (2019). *et al*. Aging-like Spontaneous Epigenetic Silencing Facilitates Wnt Activation, Stemness, and Braf(V600E)-Induced Tumorigenesis. Cancer Cell.

[22] Sato T, Yamaguchi A, Goi T, Hirono Y, Takeuchi K, Katayama K (2004). Heparanase expression in human colorectal cancer and its relationship to tumor angiogenesis, hematogenous metastasis, and prognosis. J Surg Oncol.

[23] Nobuhisa T, Naomoto Y, Ohkawa T, Takaoka M, Ono R, Murata T (2005). Heparanase expression correlates with malignant potential in human colon cancer. J Cancer Res Clin Oncol.

[24] Shen H, Yang J, Huang Q, Jiang MJ, Tan YN, Fu JF (2015). Different treatment strategies and molecular features between right-sided and left-sided colon cancers. World J Gastroenterol.

[25] Imperial R, Ahmed Z, Toor OM, Erdogan C, Khaliq A, Case P (2018). Comparative proteogenomic analysis of right-sided colon cancer, left-sided colon cancer and rectal cancer reveals distinct mutational profiles. Mol Cancer.

[26] Franke AJ, Skelton WP, Starr JS, Parekh H, Lee JJ, Overman MJ (2019). Immunotherapy for Colorectal Cancer: A Review of Current and Novel Therapeutic Approaches. J Natl Cancer Inst.

[27] Liu M, Liu Q, Yuan Y, Li S, Dong Y, Liang L *et al*. Heparanase (HPSE) Associates with the Tumor Immune Microenvironment in Colorectal Cancer. *Processes* 2021; 9.

[28] Gingis-Velitski S, Zetser A, Flugelman MY, Vlodavsky I, Ilan N (2004). Heparanase induces endothelial cell migration via protein kinase B/Akt activation. J Biol Chem.

[29] Fux L, Feibish N, Cohen-Kaplan V, Gingis-Velitski S, Feld S, Geffen C (2009). Structure-function approach identifies a COOH-terminal domain that mediates heparanase signaling. Cancer Res.

[30] Boyango I, Barash U, Fux L, Naroditsky I, Ilan N, Vlodavsky I (2018). Targeting heparanase to the mammary epithelium enhances mammary gland development and promotes tumor growth and metastasis. Matrix Biol.

[31] Massague J (2004). G1 cell-cycle control and cancer. Nature.

[32] Murray AW (2004). Recycling the cell cycle: cyclins revisited. Cell.

[33] van den Heuvel S, Dyson NJ (2008). Conserved functions of the pRB and E2F families. Nat Rev Mol Cell Biol.

[34] Malumbres M, Barbacid M (2009). Cell cycle, CDKs and cancer: a changing paradigm. Nat Rev Cancer.

[35] Shanmugasundaram K, Block K, Nayak BK, Livi CB, Venkatachalam MA, Sudarshan S (2013). PI3K regulation of the SKP-2/p27 axis through mTORC2. Oncogene.

[36] Team RC. R: A language and environment for statistical computing. R Foundation for Statistical Computing, Vienna, Austria, 2020.

[37] Silva TC, Colaprico A, Olsen C, D’Angelo F, Bontempi G, Ceccarelli M (2016). TCGA Workflow: Analyze cancer genomics and epigenomics data using Bioconductor packages. F1000Res.

[38] Marisa L, de Reynies A, Duval A, Selves J, Gaub MP, Vescovo L (2013). Gene expression classification of colon cancer into molecular subtypes: characterization, validation, and prognostic value. PLoS Med.

[39] Love MI, Huber W, Anders S (2014). Moderated estimation of fold change and dispersion for RNA-seq data with DESeq2. Genome Biol.

[40] Ritchie ME, Phipson B, Wu D, Hu Y, Law CW, Shi W (2015). limma powers differential expression analyses for RNA-sequencing and microarray studies. Nucleic Acids Res.

[41] Schneider CA, Rasband WS, Eliceiri KW (2012). NIH Image to ImageJ: 25 years of image analysis. Nat Methods.

[42] Livak KJ, Schmittgen TD (2001). Analysis of relative gene expression data using real-time quantitative PCR and the 2(-Delta Delta C(T)) Method. Methods.

[43] Liu M, Xu X, Peng K, Hou P, Liu Q, Yu Y (2021). The role of HPSE in BRAF V600E-mutant colorectal cancer. J Clin Oncol.

