# Supplementary material for: Heparanase modulates the prognosis and development of BRAF V600E-mutant colorectal cancer by regulating AKT/p27Kip1/Cyclin E2 pathway
Source: Oncogenesis. 2022 Sep 21;11(1):58. doi: 10.1038/s41389-022-00428-0 (PMC9492760; doi:10.1038/s41389-022-00428-0)
Supplement: Supplementary file 1 — Table S1, 2, 4 and Figure S1–5 [file 41389_2022_428_MOESM1_ESM.pdf]

Supplementary Information

This file includes three supplementary tables (Table S1, 2, 4) and five supplementary figures (Figure S1-5).

Table S1. Clinical characteristics of patients retrieved from public data.

Variables	TCGA				GSE39582			
	N (%)	B-MT (%)	B-WT (%)	*P*	N (%)	B-MT (%)	B-WT (%)	*P*
Overall	525 (100.0)	64 (12.2)	461 (87.8)		510 (100.0)	51 (10.0%)	459 (90.0%)	
Gender								
Female	250 (47.6)	39 (60.9)	211 (45.8)	0.032	233 (45.7)	34 (66.7)	199 (43.4)	0.003
Male	275 (52.4)	25 (39.1)	250 (54.2)		277 (54.3)	17 (33.3)	260 (56.6)	
Age (years)								
Mean (SD)	65.91 (12.94)	70.16 (13.84)	65.32 (12.71)	0.005	66.93 (13.08)	75.61 (7.78)	65.96 (13.20)	<0.001
Location								
Left	279 (55.4)	9 (15.5)	270 (60.5)	<0.001	303 (59.4)	7 (13.7)	296 (64.5)	<0.001
Right	225 (44.6)	49 (84.5)	176 (39.5)		207 (40.6)	44 (86.3)	163 (35.5)	
Stage								
I	91 (17.4)	11 (17.2)	80 (17.4)	0.006	37 (7.3)	2 (3.9)	35 (7.6)	0.493
II	195 (37.2)	35 (54.7)	160 (34.8)		246 (48.2)	22 (43.1)	224 (48.8)	
III	160 (30.5)	15 (23.4)	145 (31.5)		167 (32.7)	21 (41.2)	146 (31.8)	
IV	78 (14.9)	3 (4.7)	75 (16.3)		60 (11.8)	6 (11.8)	54 (11.8)	
MMR								
dMMR	71 (14.2)	42 (70.0)	29 (6.6)	<0.001	72 (15.6)	31 (68.9)	41 (9.8)	<0.001
pMMR	428 (85.8)	18 (30.0)	410 (93.4)		391 (84.4)	14 (31.1)	377 (90.2)	
KRAS								
MT	222 (42.3)	6 (9.4)	216 (46.9)	<0.001	204 (40.0)	0 (0.0)	204 (44.4)	<0.001
WT	303 (57.7)	58 (90.6)	245 (53.1)		306 (60.0)	51 (100.0)	255 (55.6)	

The Chi-square test was used to analyze categorical variables. The student's t test was used to analyze continuous variables. B-MT, *BRAF* V600E mutant type; B-WT, *BRAF*

wild type. MT: mutant type; WT: wild type.

Table S2. Clinical characteristics of frozen tissue samples from 44 patients with colorectal cancer.

Variables	N (%)	*BRAF* V600E MT (%)	*BRAF* WT (%)	*P*
Overall	44 (100.0)	6 (13.6)	38 (86.4)	
Gender				
Female	13 (29.5)	2 (33.3)	11 (28.9)	1
Male	31 (70.5)	4 (66.7)	27 (71.1)	
Age (years)				
Mean (SD)	65.25 (11.43)	68.00 (5.59)	64.82 (12.09)	0.532
Location				
Left	34 (77.3)	2 (33.3)	32 (84.2)	0.025
Right	10 (22.7)	4 (66.7)	6 (15.8)	
Stage				
IV	44 (100.0)	6 (100.0)	38 (100.0)	NA
MMR				
pMMR	42 (95.5)	5 (83.3)	37 (97.4)	0.632
dMMR	2 (4.5)	1 (16.7)	1 (2.6)	

The Chi-square test was used to analyze categorical variables. The student's t test was used to analyze continuous variables.

Table S4. Primer sequences used for RT-PCR.

Primers	Sequence (5’-3’)
HPSE-F	5′-CTCTATGGTCCTGATGTTGGTC-3′
HPSE-R	5′-AAATCTTCCTTGGTAGCAGTCC-3′
GAPDH-F	5′-CCCATCACCATCTTCCAGG-3′
GAPDH-R	5′-GAGATGATGACCCTTTTGGC-3′

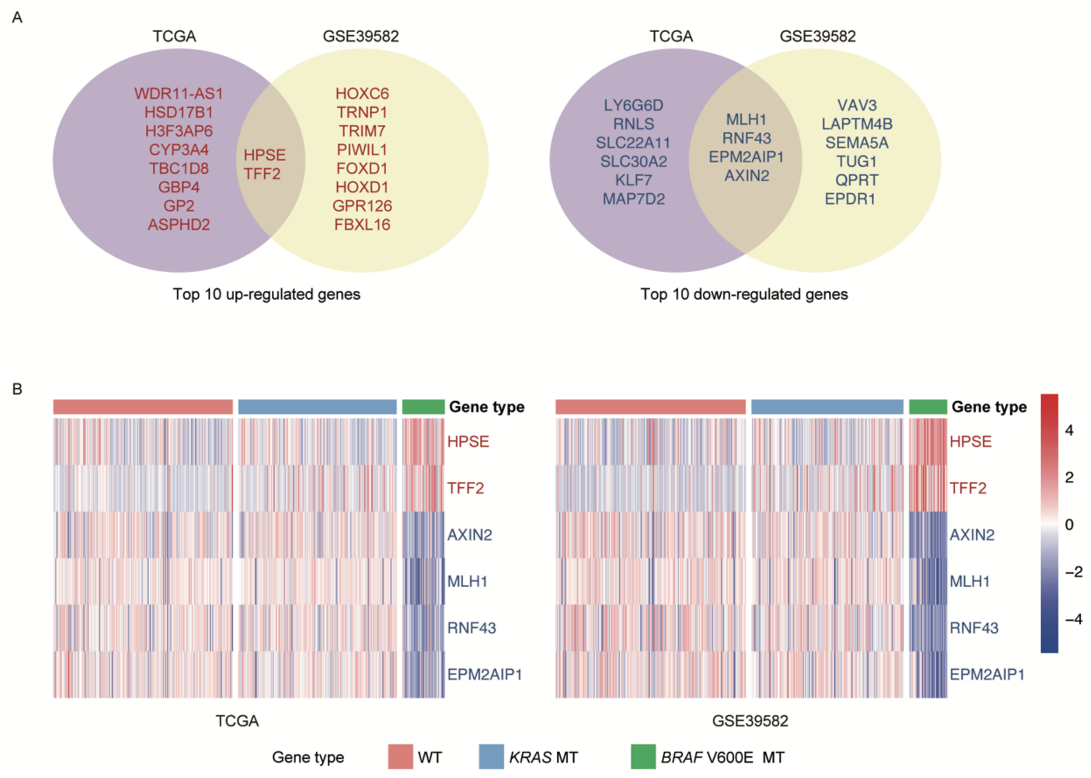

Figure S1. Differentially expressed genes (DEGs) between *BRAF* V600E-mutant and wild-type colorectal cancer. (A) Venn diagrams of the top ten differentially expressed genes in the two datasets. **(B)** Heat maps of the top six differentially expressed genes in CRC samples with

different gene types. R pheatmap package was used to generate heat maps. WT: *KRAS/BRAF* wild type; *KRAS* MT: *KRAS* mutant type; *BRAF* V600E MT: *BRAF* V600E mutant type.

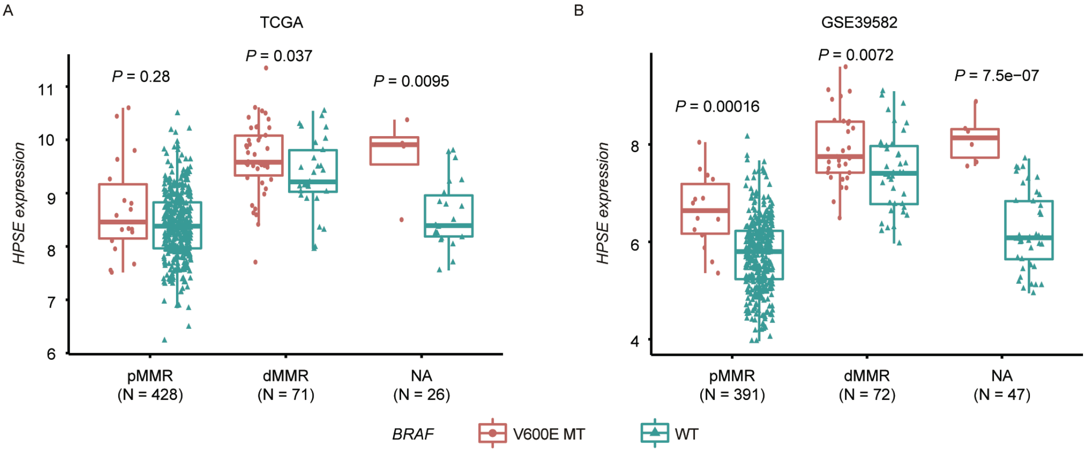

Figure S2. *HPSE* expression levels in *BRAF* V600E-mutant and wild-type CRC patients within different subgroups of MMR status in TCGA (A) and GSE39582 (B). Dots represent samples, center lines indicate median, box plots indicate the quartiles, and bars represent 95% confidence intervals. Box plots were generated using ggplot2 package and ggpubr package. Wilcoxon rank-sum test was used to determine *P* values. NA, not available.

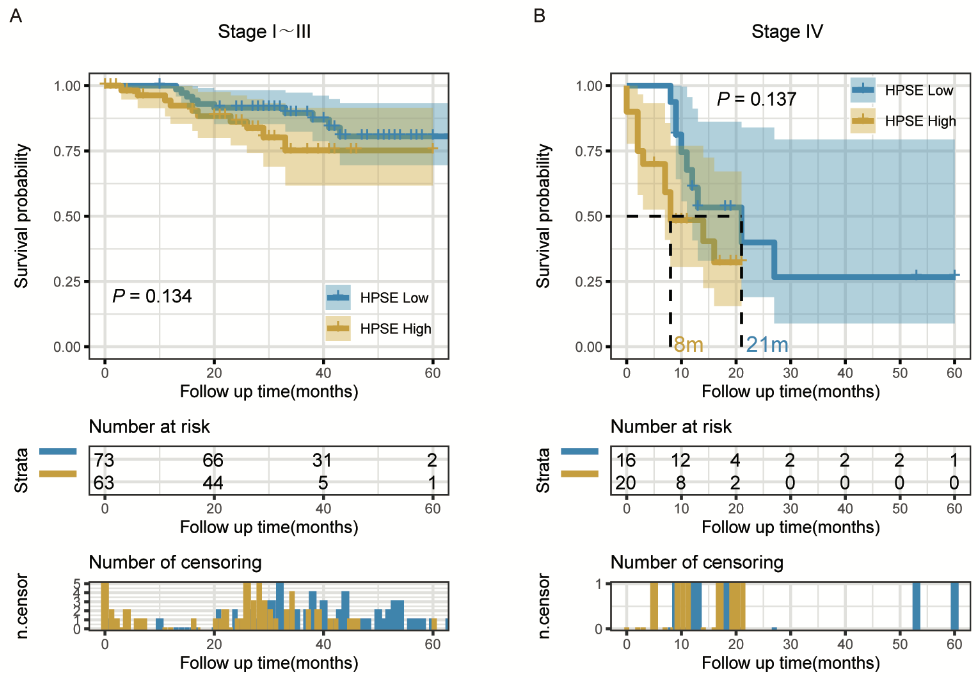

Figure S3. Survival curves of HPSE high and low groups in *BRAF* V600E-mutant CRC at different stages. (A) Kaplan-Meier survival curves for the stage I~III subgroup. **(B)** Kaplan-Meier survival curves for the stage IV subgroup. The Log-rank test was used to determine *P* values. Shaded regions around the curves indicate 95% confidence intervals. The number of patients at risk and censoring are presented below the survival curve.

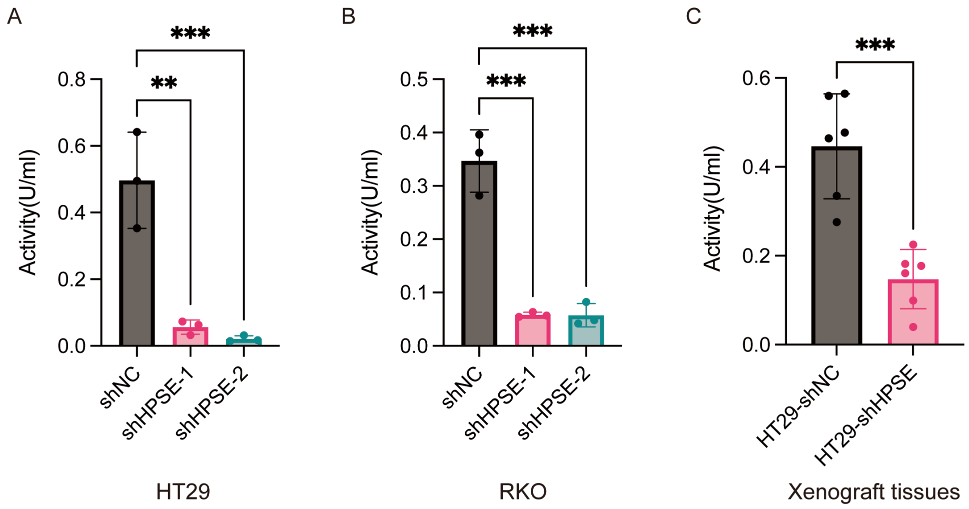

Figure S4. Silencing *HPSE* expression suppresses HPSE enzymatic activity in BRAF V600E-mutant CRC cells and xenografts. The enzymatic activity of HPSE was measured in HT29 **(A)**, RKO**(B)**, HT29-derived xenograft tissues**(C)**. Data are presented as the mean ± standard deviation. One-way ANOVA analysis and Tukey's test were used for multiple comparisons. *** P<0.001, ** P<0.01.

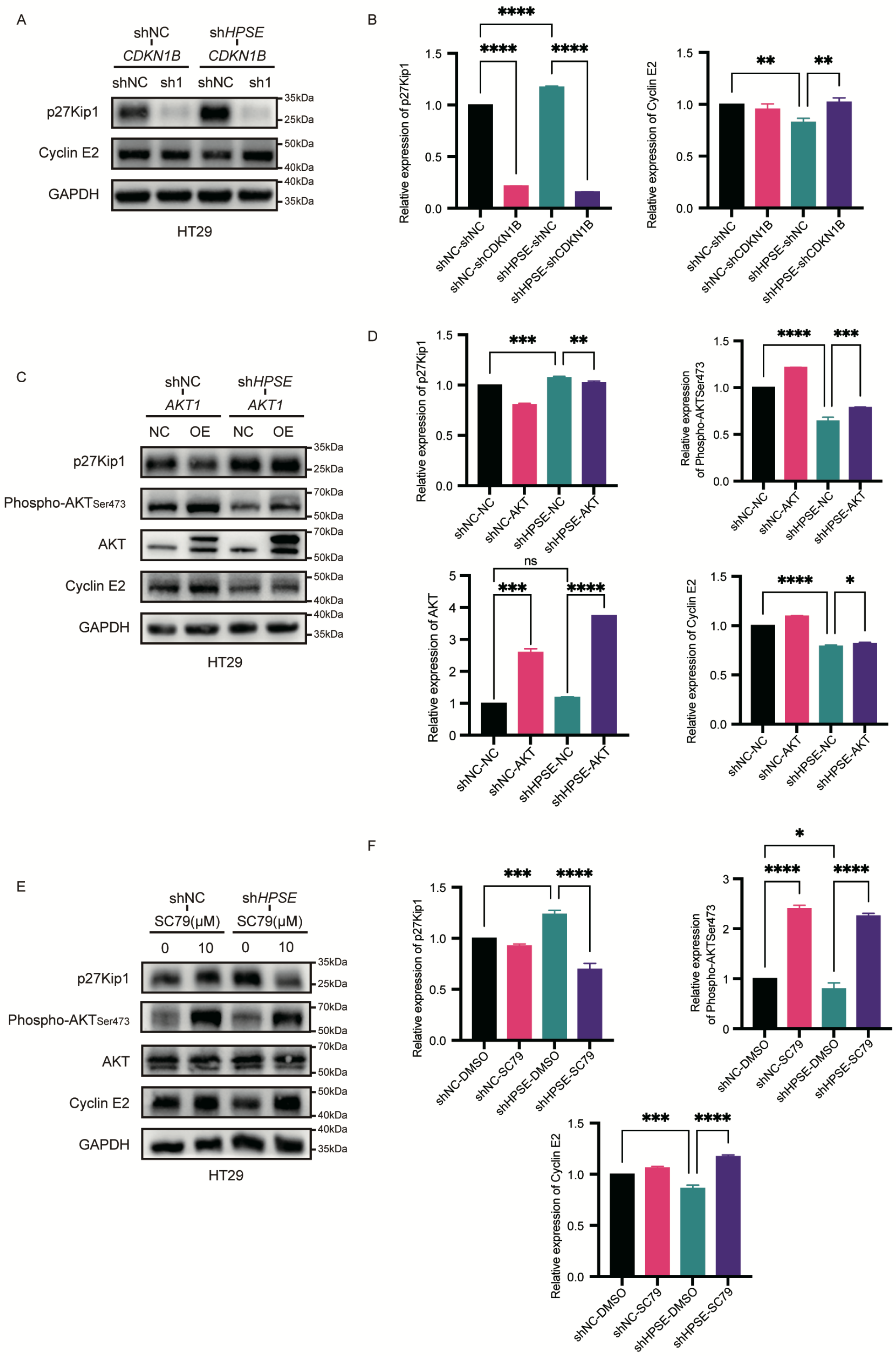

Figure S5. Silencing *HPSE* expression downregulates Cyclin E2 expression in *BRAF* V600E-mutant CRC cells through AKT/p27Kip1 pathway. Expression levels of key molecules at protein level after silencing of *CDKN1B* (**A**), overexpression of *AKT1* (**C**), and

exogenous activation of the AKT phosphorylation (**E**) in *HPSE*-knockdown and control cells. In the group treated with SC79, cells were incubated with DMSO/SC79 (10 μM) for 72h.The same trend was observed in three independent repeated experiments. Representative results are shown. NC, negative control. OE, overexpression. **(B) (D) (F)** Results of semi-quantitative analysis of bands in **(A), (C), (E)** are shown respectively. Data are presented as the mean ± standard deviation. One-way ANOVA analysis and Tukey's test were used for multiple comparisons. **** $P<0.0001$, *** $P<0.001$, ** $P<0.01$, * $P<0.05$, ns $P>0.05$.
